# Supplementary material for: A versatile nuclei extraction protocol for single nucleus sequencing in non-model species–Optimization in various Atlantic salmon tissues
Source: PLoS One. 2023 Sep 7;18(9):e0285020. doi: 10.1371/journal.pone.0285020 (PMC10484441; doi:10.1371/journal.pone.0285020)
Supplement: S1 File — (PDF) [file pone.0285020.s001.pdf]

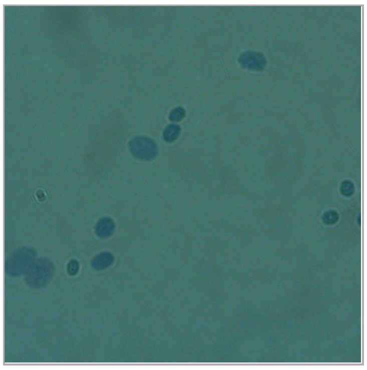

VERSION 2

SEP 27, 2022

OPEN ACCESS

DOI:
dx.doi.org/10.17504/protocols.io.261genwm7g47/v2

Protocol Citation: Rose Ruiz Daniels, Richard S Taylor, Ross Dobie, Sarah Salisbury, Emily Clark, Dan Macqueen, Diego Robledo 2022. A versatile nuclei extraction protocol for single nucleus sequencing in non model species – optimization in various Atlantic salmon tissues.. **protocols.io** https://dx.doi.org/10.17504/protocols.io.261genwm7g47/v2Version created by Rose Ruiz Daniels

MANUSCRIPT CITATION:
Taylor, R, Ruiz Daniels, R, Dobie, R, Naseer, S, Clark, TC, Henderson, NC, Boudinot, P, Martin, SAM & Macqueen, D 2022, 'Single cell transcriptomics of Atlantic salmon (Salmo salar L.) liver reveals cellular heterogeneity and immunological responses to challenge by Aeromonas salmonicida', Frontiers in Immunology, pp. 1-17. https://doi.org/10.3389/fimmu.2022.984799

A versatile nuclei extraction protocol for single nucleus sequencing in non model species – optimization in various Atlantic salmon tissues. V.2

Rose Ruiz Daniels[1], Richard S Taylor[1], Ross Dobie[2], Sarah Salisbury[1], Emily Clark[1], Dan Macqueen[1], Diego Robledo[1]

[1]The Roslin Institute and Royal (Dick) School of Veterinary Studies, The University of Edinburgh, Edinburgh EH25 9RG, UK;
[2]Centre for Inflammation Research, The Queen’s Medical Research Institute, Edinburgh BioQuarter, University of Edinburgh, EH16 4TJ, UK

The Roslin Institute

Rose Ruiz Daniels

DISCLAIMER

DISCLAIMER – FOR INFORMATIONAL PURPOSES ONLY; USE AT YOUR OWN RISK

The protocol content here is for informational purposes only and does not constitute legal, medical, clinical, or safety advice, or otherwise; content added to protocols.io is not peer reviewed and may not have undergone a formal approval of any kind. Information presented in this protocol should not substitute for independent professional judgment, advice, diagnosis, or treatment. Any action you take or refrain from taking using or relying upon the information presented here is strictly at your own risk. You agree that neither the Company nor any of the authors, contributors, administrators, or anyone else associated with protocols.io, can be held responsible for your use of the information contained in or linked to this protocol or any of our Sites/Apps and Services.

License: This is an open access protocol distributed under the terms of the [Creative Commons Attribution License], which permits unrestricted use, distribution, and reproduction in any medium, provided the original author and source are credited

Protocol status: Working
We use this protocol and it's working

Created: Jul 25, 2022

Last Modified: Sep 27, 2022

PROTOCOL integer ID: 67518

Keywords: snRNA-seq , aquaculture, non-model species, nuclei

ABSTRACT

Single cell RNA sequencing has rapidly become a standard tool for profiling transcriptomic diversity across thousands of cells (Linnarsson and Teichmann, 2016), and is now being applied to a large diversity of species and tissues. The main limitation of this technology is that it requires the isolation of live cells from fresh tissue, severely restricting its applicability. As a result, single nuclei RNA sequencing (snRNA-seq), which consists of sequencing the RNA of only the nuclei of cells rather than of the whole cell, has been commonly adopted since it allows samples to be stored for several months prior to processing while yielding comparable results to whole cell sequencing (Kulkarni, et al., 2019; Slyper et al. 2021). A critical challenge for snRNA-seq is the successful extraction of high quality nuclei. This has spurred the recent publication of a number of dissociation protocols for nuclei extraction (Drokhlyansky et al. 2020; Eraslan et al. 2021; Melms et al 2021), however, these have largely been optimized for model species such as humans, and more and more single nuclei is being adopted in non-model species.

Here we present a robust protocol that enables the extraction of nuclei from frozen tissue adapted from those shown to work in different tissue types, such as human skin (Drokhlyansky et al. 2020; Eraslan et al. 2021; Melms et al 2021). Our protocol has been used to successfully extract nuclei from an array of different Atlantic salmon (Salmo salar) tissues including liver, skin, fin, spleen, head kidney and gill as well as in other species such as sole (Solea solea) nose and gonad, rabbit (Oryctolagus cuniculus) nasal tissue and nurse shark (Ginglymostoma cirratum) spleen. We present the protocol as applied to fin and skin as these are particularly challenging tissues to work with given their toughness and the presence of hard tissue (e.g., scales and bones), connective tissue and fat deposits. We include notes throughout the protocol so that the reader can optimise it for a variety of tissue types. While the protocol has been optimised to work with the Chromium 10x platform, the most commonly used high throughput microfluidic device, but can be used successfully for the extraction of nuclei for other platforms and applications. The aim of this protocol is to capture 7,000 nuclei per single-nuclei RNA sequencing library using the Chromium Single Cell 3' Reagent Kits v2 or v3 (10X Genomics). Given its utility for isolating nuclei from difficult to dissociate tissue types, we anticipate that this protocol will be broadly applicable for snRNA-seq of non-model organisms and unconventional tissue types.

ATTACHMENTS

hc6dbbap7.docx   figure 1 ressults.docx

GUIDELINES

References:

CITATION

Drokhlyansky E, Smillie CS, Van Wittenberghe N, Ericsson M, Griffin GK, Eraslan G, Dionne D, Cuoco MS, Goder-Reiser MN, Sharova T, Kuksenko O, Aguirre AJ, Boland GM, Graham D, Rozenblatt-Rosen O, Xavier RJ, Regev A (2020). The Human and Mouse Enteric Nervous System at Single-Cell Resolution.. Cell.

LINK

https://doi.org/10.1016/j.cell.2020.08.003

Eraslan, G, Drokhlyansky E, Anand S, Subramanian A, Fiskin, E, Slyper M, Wang J, Wittenberghe N. Van; Rouhana, J.M.; Waldman, J.; et al. Single-nucleus cross-tissue molecular reference maps to decipher disease gene function. Science 2022.https://doi.org/10.1126/science.abl429090

Kulkarni, A.; Anderson, A.G.; Merullo, D.P.; Konopka, G. Beyond bulk: a review of single cell transcriptomics methodologies and applications. Curr. Opin. Biotechnol. 2019. https://doi.org/10.1016/j.copbio.2019.03.001.

CITATION

Linnarsson S, Teichmann SA (2016). Single-cell genomics: coming of age.. Genome biology.

LINK

https://doi.org/10.1186/s13059-016-0960-x

CITATION

Slyper M, Porter CBM, Ashenberg O, Waldman J, Drokhlyansky E, Wakiro I, Smillie C, Smith-Rosario G, Wu J, Dionne D, Vigneau S, Jané-Valbuena J, Tickle TL, Napolitano S, Su MJ, Patel AG, Karlstrom A, Gritsch S, Nomura M, Waghray A, Gohil SH, Tsankov AM, Jerby-Arnon L, Cohen O, Klughammer J, Rosen Y, Gould J, Nguyen L, Hofree M, Tramontozzi PJ, Li B, Wu CJ, Izar B, Haq R, Hodi FS, Yoon CH, Hata AN, Baker SJ, Suvà ML, Bueno R, Stover EH, Clay MR, Dyer MA, Collins NB, Matulonis UA, Wagle N, Johnson BE, Rotem A, Rozenblatt-Rosen O, Regev A (2020). A single-cell and single-nucleus RNA-Seq toolbox for fresh and frozen human tumors.. Nature medicine.

LINK

https://doi.org/10.1038/s41591-020-0844-1

MATERIAL

Noyes Spring Scissors - Tungsten Carbide **Fine Science Tools Catalog #15514-12**

Tungsten Carbide Straight 11.5 cm Fine Scissors **Fine Science Tools Catalog #14558-11**

40 µm Falcon™ Cell Strainers **Fisher Scientific Catalog #08-771-2**

Corning™ Falcon™ Test Tube with 35µm Cell Strainer Snap Cap **Corning Catalog #352235**

pluriStrainer Mini 20 µm (Cell Strainer) **pluriSelect Catalog #43-10020-50**

X500 Eppendorf DNA LoBind Tubes, 1.5ml, PCR clean

Cryotube

6-well tissue culture plate (Stem Cell Technologies)

Falcon tubes 15 ml (Corning)

INCYTO C-Chip™ Disposable Hemacytometers **VWR International Catalog #82030-468**

SAMPLING AND STORAGE FOR NUCLEAR ISOLATION

Animals must be appropriately euthanized and immediately processed. Approximately ~ 60 mg of salmonid tissue is placed in one clearly labelled cryotube and immediately flash frozen in liquid nitrogen. **This step is critical**. The tissue must be preserved as fast as possible for optimal results. In the absence of liquid nitrogen, samples can be frozen in dry ice. Samples can be stored at -80 °C for up to a year prior to use. Older samples might still yield viable nuclei but this would need to be tested.

REAGENTS

All reagents should be chilled on ice prior to use.

2X stock of salt-Tris solution makes 10 mL :

Stocks:

NaCl: NaCl (5 M) RNase-free **Thermo Fisher Scientific Catalog #AM9759**

Tris-HCl pH 7.5:

UltraPure™ 1 M Tris-HCl Buffer, pH 7.5 **Thermo Fisher Catalog #15567027**

CaCl2:

Calcium chloride 1 M in aqueous solution **VWR International Catalog #97062-820**

MgCl2:

Magnesium chloride solution for molecular biology (1.00 M) **Merck MilliporeSigma (Sigma-Aldrich) Catalog #M1028**

Nuclease-free water:

Water for biotechnology nuclease-free sterile **VWR International Catalog #97062-794**

A	B	C
Stock solution (see above)	Volume	Final concentration
NaCl	292 ul	146 mM
Tris-HCL10	100 ul	10 mM
CaCl2	10 ul	1 mM
MgCl2	210 ul	21 mM
Nuclease-free water	9388 ml	

The following buffers contain RNAase inhibitor

Protector RNase Inhibitor **Merck MilliporeSigma (Sigma-Aldrich) Catalog #3335399001**

- It is important to use the correct RNAse inhibitor as it can negatively affect library prep, check with the sequencing platform before using another type of RNAse.
- Do not add RNAse until right before nuclear extraction.
- RNAse inhibitor does not need to be used to test nuclear extractions, but it should added for sequencing runs.

1X ST buffer solution (**ST**) - 10 mL :

Dilute 2x ST in ultrapure nuclease-free water (1:1)

A	B	C
Stock Solution	Volume	Final concentration
2X ST	3 ml	
Ultrapure nuclease free water	3 ml	
RNAse inhibitor	6ul µl (240 U)	40 Uml

Make fresh and chill prior to use, add RNAnase inhibitor right before nuclear isolation. RNAase inhibitor amount can up upped if it's an RNAse Rich tissue, up to 500 U per ml instead, tissue spends very little time in this buffer and is chilled at all time, which is why the amount of RNAase inhibitor can be lower.

Working solution (**TST**) – 4 mL:

1% Tween-20:

Tween-20 **Merck MilliporeSigma (Sigma-Aldrich) Catalog #P-7949**

2% BSA:

Bovine Serum Albumin (20 mg/mL) Molecular Biology Grade **New England Biolabs Catalog #B9000S**

A	B	C
Stock solution	Volume	Final concentration
2X ST buffer	2 ml	
1% Tween-20	120 µl	
2% BSA	20 µl	
Nuclease-free water	1840 µl	
RNAse inhibitor	20 µl (800 U)	200 Uml

Make fresh and chill prior to use, add RNAase inhibitor right before nuclear isolation .Dilute the Tween from 10% in stock solution with nfH2O before making the buffer. RNAnase inhibitor amount can be upped if it's an RNAase rich tissue up to 1000 U per ml instead, the nuclear isolation will happen in this buffer so its more critical in here.

PBS+0.02 BSA (**PBS+BSA**) – 1 mL:

A	B	C
Stock solution	Volume	Final concentration
Ultra-pure molecular grade PBS	1970 µl	
2% BSA **	20 µl	
RNAse inhibitor	10 µl	200 Uml

** can top this up this to 2% BSA if the cells are clumping or look degraded .RNAase inhibitor is the most critical in this step as the nuclei will be in this buffer the longest can use up to 1000 U per ml.

BEFORE START INSTRUCTIONS

Sampling and storage for nuclear isolation.
Animals must be appropriately euthanized and immediately processed. Approximately ~ 60 mg of tissue is placed in one clearly labelled cryotube and immediately flash frozen in liquid nitrogen. **This step is critical**. The tissue must be preserved as fast as possible for optimal results. In the absence of liquid nitrogen, samples can be frozen in dry ice. Samples can be stored at -80 °C for up to a year prior to use. Older samples might still yield viable nuclei but this would need to be tested.

All reagents should be chilled on ice prior to use.
Samples should be kept frozen on dry ice until immediately before nuclei isolation, and all sample-handling steps should be performed on ice.
The centrifuge should be pre chilled at 4 °C .

All reagents are given for 2 nuclear isolations.
Amounts of buffer especially those that contain RNase should be adjusted appropriately for each experiment prepared prior and RNase added immediately before use.

Before using this prep for library preparation do a trial run.
Recommended to do a trial especially on a new tissue type to adjust different parameters without adding RNase. Once parameters are adjusted such as mincing times, filter size and dilution in to final buffer in order to get good quality nuclei.

Nucleus isolation workflow for ST-based buffers

30m

1

Note

Samples should be kept frozen on dry ice until immediately before nuclei isolation, and all sample-handling steps should be performed On ice. The centrifuge should be pre-chilled at 4 °C.

On ice, place a piece of frozen tissue into one well of a 6-well tissue culture plate with 1 mL TST.

Note

If the sample is stuck to the cryotube, remove using tweezers, preferably while still in dry ice, and place immediately into the culture plate with TST. If the sample needs processing for examples cutting this is best done on dry ice. This is avoided by processing the sample prior to flash freezing.

2 On ice, mince tissue initially using Tungsten Carbide scissors for 00:00:30 and then with Noyes Spring Scissors Noyes Spring Scissors - Tungsten Carbide **Fine Science Tools Catalog #15514-12** for a total of 00:10:00. 10m

Note

This step is only necessary for fin, skin or similar hard tissues, for softer tissues just use spring scissors for 00:10:00.

2.1 00:05:00 into the mincing gently pipette up and down with a p1000 pipette using a low retention filtered tip. The time in the dissociation buffer is critical. See image for how to assess the timing is correct by looking at your nuclei. 5m

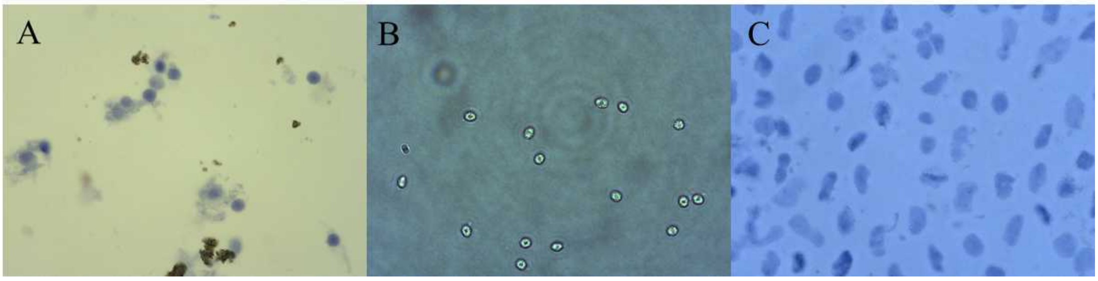

Image from different dissociation trials in Atlantic salmon tissues x40 magnification stained with trypan blue. **A**. Head kidney nuclei not had sufficient time in dissociation buffer, will clog microfluidic device. **B**. Blood nuclei perfectly dissociated minimal clumping ideal for sequencing. **C**. Liver nuclei to long in dissociation buffer, nuclear membrane started to degrade. Can still be sequenced but not ideal. Note when staining nuclei with trypan blue asses nuclear quality as soon as possible as the nuclei will quickly degrade when not on ice.

3 Pass lysate through a 40 µm cell strainer .

3.1 Add a further 1 mL of TST to the cell strainer immediately.

3.2 Add 3 mL of freshly prepared ST buffer to the lysate.

3.3 Add the 5 mL of lysate to a marked 15 ml falcon tube (Corning) on ice.

4 Centrifuge at 500 x g, 4°C, 00:05:00 in a swinging bucket centrifuge. 5m

5 Resuspend the pellet gently using a p1000 pipette in PBS-BSA.

Note

Resuspension volume depends on the size of the pellet, usually within the range of 100 µL - 1000 µL (1 mL if there are many nuclei). For skin and fin, 400 µL is recommended.

6 Filter the nucleus solution a second time.

Note

The size of the filter is tissue dependant, e.g. for tissues such as liver and head kidney a 40 µm Falcon™ cell strainer will suffice, whereas for gill, a 30 µm filter would be better giving the higher amount of tough debris that could clog the microfluidic device. In addition, for harder tissues that produce a lot of debris such as fin and skin (this is due to the presence of fat layers and scales in skin and the presence of bones in the fin) then 20 µm is recommended. The lysate may not pass through at once, pipetting very gently up and down with a wide bore pipette can help it through.

7 Count the nuclei using a C-chip disposable haemocytometer.

Note

In this step, it is also possible to visualise the nuclei and ascertain the level of debris present as well as the integrity of the nuclear membrane.

8 The nuclei are also counted using a Bio-Rad TC20 to confirm results from the disposable haemocytometer and to count the proportion of viable cells.

Note

Nuclei are identified as “dead”, therefore a good nuclei isolation will have a small percentage of live cells. 1-4% of live cells is ideal, but below 12% is acceptable. High proportions of live cells indicates incomplete nuclear isolation and could be an indication of high amounts of debris or insufficient lysis time.

9 Load the nucleus suspension into a Chromium Chip and into the Chromium Controller, aiming to recover 7,000 nuclei as per 10x recommendations with a concentration of between 700 to 1200 nuclei per µl.

Note

In the case of some tissues such as fin, readjust the target recovery to 5000 especially with juvenile fish or tissues such as fin and skin as nuclei yields are on the low side.

10

11
