# Supplementary material for: A versatile nuclei extraction protocol for single nucleus sequencing in non-model species–Optimization in various Atlantic salmon tissues
Source: PLoS One. 2023 Sep 7;18(9):e0285020. doi: 10.1371/journal.pone.0285020 (PMC10484441; doi:10.1371/journal.pone.0285020)
Supplement: Attachment — Submitted filename: Ruiz Daniels manuscript_Plos one 2023 review letter_R1.pdf [file pone.0285020.s004.pdf]

To the Editor,

I believe that the changes made by the authors of the Ruiz Daniels manuscript are sufficient and would support the decision of the editor to accept this paper.

Sincerely,

Steven Weicksel
