# Supplementary material for: A versatile nuclei extraction protocol for single nucleus sequencing in non-model species–Optimization in various Atlantic salmon tissues
Source: PLoS One. 2023 Sep 7;18(9):e0285020. doi: 10.1371/journal.pone.0285020 (PMC10484441; doi:10.1371/journal.pone.0285020)
Supplement: Attachment — Submitted filename: Ruiz Daniels manuscript_Plos one 2023 review letter.docx [file pone.0285020.s002.docx]

In this manuscript the authors describe a method of nuclei purification that increases yield and quality of the collected sample over previous methods. The authors also present data comparing the end result of their method to the previously used method. Overall, the steps of the manuscript are clear, and the protocol meets its claim as being better suited than the previous method.

Major issues:

Though the data is convincing, I’m not sure that the pervious method of nuclei purification was a truly suitable method for the types of tissues described here, ie. frozen and tough tissue samples. The citation given links to methods used for soft laboratory derived tissues. Considering that I wonder what the justification of using the previous approach would be. Searching the methodologies of projects purifiy nuclei from hard to access tissues, these also use mechanical methods (such as grinding and cutting the tissue). These methodologies however maybe more appropriate as points of comparison.

Minor issues:

The second paragraph of the introduction has a lot of “should” “could” “would” “anticipate”. Ideal the paragraph would read more like the first and be more direct and succinct.

Using a glycerol cushion to remove debris instead of a filter.

Not sure why the whole protocol is rehashed in the “testing the protocol” section. In this section it seems that it would be most appropriate to explain what experiment was set up or just scrap it and have a results section.

Citations were done improperly, please check the Plos One guidelines.

It is hard to determine what the actual changes in the protocol are actually doing and whether or not this group is just much better than others at collecting nuclei. Part of this I believe is the structure of the article. Though this is a protocol, there still should be

- Line 21 – typo, word missing
- Line 131 – clarify what is adjusted. This statement about the time isn’t very clear. It might be clearer to state that dissociation takes up to 10 minutes. “First 5 minutes of mincing and then pipetting for up to 5 minutes checking every minute for completion of nuclei dissociation.”
- Line 132 – two sentences
- Line 140 – to be more universal, should the centrifuge time just be set to 10 minutes.
- Line 163 – seems that this should be stated in the discussion or at the least a sub step of step 7. This also seems to be a specific type of equipment that isn’t available to everyone, where as a haemocytometer is more readily available.
- Line 180 – should be reworded to not “hang” on the citation or state “from Drokhlyansky and colleagues^4^”. There are several places in the manuscript this is done and there are ways around it.
- Line 180-206 – It sounds like you tested your version to the previous published version. I believe that you should just state that and reference the changes you made. I’m not sure why the whole protocol is restated.
- Figure 2 - Not sure why figure two isn’t just a bar graph. Using these shapes indicates that there is other information here. If that is the case, it should be explain, otherwise standard bar graphs should be used.
- Figure 2 – typo in the legend.
